# *Vital Signs:* Health Disparities in Hemodialysis-Associated *Staphylococcus aureus* Bloodstream Infections — United States, 2017–2020

**DOI:** 10.15585/mmwr.mm7206e1

**Published:** 2023-02-10

**Authors:** Brian Rha, Isaac See, Lindsay Dunham, Preeta K. Kutty, Lauren Moccia, Ibironke W. Apata, Jennifer Ahern, Shelley Jung, Rongxia Li, Joelle Nadle, Susan Petit, Susan M. Ray, Lee H. Harrison, Carmen Bernu, Ruth Lynfield, Ghinwa Dumyati, Marissa Tracy, William Schaffner, D. Cal Ham, Shelley S. Magill, Erin N. O’Leary, Jeneita Bell, Arjun Srinivasan, L. Clifford McDonald, Jonathan R. Edwards, Shannon Novosad

**Affiliations:** ^1^Division of Healthcare Quality Promotion, National Center for Emerging and Zoonotic Infectious Diseases, CDC; ^2^Division of Renal Medicine, Emory University School of Medicine, Atlanta, Georgia; ^3^University of California, Berkeley, Berkeley, California; ^4^California Emerging Infectious Program, Oakland, California; ^5^Connecticut Department of Public Health; ^6^Division of Infectious Diseases, Emory University School of Medicine, Atlanta, Georgia; ^7^University of Pittsburgh, Pittsburgh, Pennsylvania; ^8^Johns Hopkins Bloomberg School of Public Health, Baltimore, Maryland; ^9^Minnesota Department of Health; ^10^University of Rochester Medical Center, Rochester, New York; ^11^Vanderbilt University Medical Center, Nashville, Tennessee.

## Abstract

**Introduction:**

Racial and ethnic minorities are disproportionately affected by end-stage kidney disease (ESKD). ESKD patients on dialysis are at increased risk for *Staphylococcus aureus* bloodstream infections, but racial, ethnic, and socioeconomic disparities associated with this outcome are not well described.

**Methods:**

Surveillance data from the 2020 National Healthcare Safety Network (NHSN) and the 2017–2020 Emerging Infections Program (EIP) were used to describe bloodstream infections among patients on hemodialysis (hemodialysis patients) and were linked to population-based data sources (CDC/Agency for Toxic Substances and Disease Registry [ATSDR] Social Vulnerability Index [SVI], United States Renal Data System [USRDS], and U.S. Census Bureau) to examine associations with race, ethnicity, and social determinants of health.

**Results:**

In 2020, 4,840 dialysis facilities reported 14,822 bloodstream infections to NHSN; 34.2% were attributable to *S. aureus*. Among seven EIP sites, the *S. aureus* bloodstream infection rate during 2017–2020 was 100 times higher among hemodialysis patients (4,248 of 100,000 person-years) than among adults not on hemodialysis (42 of 100,000 person-years). Unadjusted *S. aureus* bloodstream infection rates were highest among non-Hispanic Black or African American (Black) and Hispanic or Latino (Hispanic) hemodialysis patients. Vascular access via central venous catheter was strongly associated with *S. aureus* bloodstream infections (NHSN: adjusted rate ratio [aRR] = 6.2; 95% CI = 5.7–6.7 versus fistula; EIP: aRR = 4.3; 95% CI = 3.9–4.8 versus fistula or graft). Adjusting for EIP site of residence, sex, and vascular access type, *S. aureus* bloodstream infection risk in EIP was highest in Hispanic patients (aRR = 1.4; 95% CI = 1.2–1.7 versus non-Hispanic White [White] patients), and patients aged 18–49 years (aRR = 1.7; 95% CI = 1.5–1.9 versus patients aged ≥65 years). Areas with higher poverty levels, crowding, and lower education levels accounted for disproportionately higher proportions of hemodialysis-associated *S. aureus* bloodstream infections.

**Conclusions and implications for public health practice:**

Disparities exist in hemodialysis-associated *S. aureus* infections. Health care providers and public health professionals should prioritize prevention and optimized treatment of ESKD, identify and address barriers to lower-risk vascular access placement, and implement established best practices to prevent bloodstream infections.

## Introduction

More than 800,000 persons in the United States live with ESKD, 70% of whom are treated with dialysis (89% hemodialysis and 11% peritoneal dialysis); 30% have a functioning kidney transplant (*1*). Race, ethnicity, and social determinants of health* affect development of ESKD (*1*–*4*). ESKD prevalence is fourfold higher among Black persons and more than twofold higher among Hispanic than among White persons (*1*), disparities which are thought to be attributable at least in part to underlying conditions such as hypertension and diabetes mellitus (*1*–*3*). Furthermore, disparities in pre-ESKD nephrology care and receipt of ESKD therapies exist for these same groups, as well as those with lower income and insurance coverage (*1**,**5**–**9*). Black persons constitute 33% of all U.S. patients receiving dialysis (*1*), but only 12% of the U.S. population (*10*).

Infections are a leading cause of morbidity and mortality in hemodialysis patients (*1*). *S. aureus* is the most commonly isolated pathogen among bloodstream infections in hemodialysis patients reported to NHSN; 40% of those infections are methicillin resistant (MRSA)^†^ (*11*). Higher rates of invasive *S. aureus* infections have been observed in dialysis patients compared with nondialysis patients (*12*).

Type of hemodialysis access is a well-established risk factor for infections; risk is highest for central venous catheters (CVCs), lower for grafts, and lowest for fistulas (*11*). Although elevated rates have been reported for both invasive MRSA infections among Black dialysis patients (*13*) and hospitalizations for dialysis-related infections among adult Black patients and older Hispanic patients (aged >60 years) (*14*), the association among hemodialysis-related infections, race and ethnicity, and social determinants of health is largely undescribed. To identify groups experiencing high numbers and risk of infections and to determine which preventive interventions should be prioritized, this study used a national facility-level reporting system and a laboratory- and population-based surveillance network to understand markers of disparities in the risk for *S. aureus* bloodstream infections in hemodialysis patients. This activity was reviewed by CDC and was conducted consistent with applicable federal law and CDC policy.^§^

## Methods

**NHSN.** *S. aureus* bloodstream infection was defined as a new positive blood culture test result reported from outpatient dialysis facilities during 2020. Dialysis facilities report bloodstream infections and vascular access in place at the time of event as well as monthly denominator data (patient-months summed from patients who received treatment during the first 2 working days of each month) by vascular access type.^¶^ Bloodstream infections and patient-months were categorized by type of vascular access: CVC, fistula, and graft or other. Facility-specific characteristics were reported through 2020 annual survey data. CDC/ATSDR SVI data from 2018 were linked to the facility at the county level (*15*).

A total of 7,097 dialysis facilities were included in this analysis. Incidences for *S. aureus* bloodstream infections were created by pooling events as the numerator and patient-months as the denominator for each vascular access type as reported by each facility. A statistical model** was used to assess potential associations between the main outcome of facility *S. aureus* bloodstream infection incidence with patient vascular access type and selected dialysis facility characteristics, including those related to infection control practices (45 variables), and SVI data (20 variables) (*15*).^††^ All analyses were conducted using SAS software (version 9.4; SAS Institute).

**EIP.** *S. aureus* bloodstream infection data among adult hemodialysis patients (aged ≥18 years) from CDC’s EIP surveillance in selected counties from seven sites (selected counties within each state) during 2017–2020 were analyzed; data on race and ethnicity were included.^§§^ Sites, case definitions, and methodology have been described previously (*12*). EIP site staff members geocoded patient addresses to U.S. Census Bureau tracts, which were then linked to selected socioeconomic status (SES) factors for these tracts (i.e., measures of poverty, crowding, and education).^¶¶^

Unadjusted *S. aureus* bloodstream infection rates were calculated for hemodialysis patients and the general population (adults not on hemodialysis) for the surveillance area. EIP *S. aureus* bloodstream infection data were used as the numerator for calculating rates. The denominators for *S. aureus* bloodstream infection rates among hemodialysis patients were obtained as follows: for each year of data, aggregated county-level denominator data on the number of hemodialysis patients (number of hemodialysis patients as of December 31 of the preceding calendar year) were obtained from USRDS. USRDS provided the data stratified by sex, age group (18–49, 50–64, and ≥65 years), race and ethnicity, and vascular access type (analyzed as CVC versus fistula or graft) for each year. The denominator for calculating *S. aureus* bloodstream infection rates among the general population was obtained by subtracting the hemodialysis population from the total population of the surveillance area. Population totals for the entire surveillance catchment area (i.e., including persons not receiving dialysis) were obtained from U.S. Census Bureau data.***

Unadjusted *S. aureus* bloodstream infection rates among hemodialysis patients were stratified by the characteristics described in the USRDS data. To handle overdispersion, negative binomial regression was performed to determine aRRs for age, race and ethnicity, sex, vascular access type, and EIP site.

## Results

**NHSN.** During 2020, 4,840 dialysis facilities (68.2% of 7,097 reporting to NHSN) reported 14,822 bloodstream infections; *S. aureus* was isolated from 5,070 (34.2%), yielding a rate of 0.1 *S. aureus* bloodstream infections per 100 patient-months. Among reported *S. aureus* bloodstream infections, 2,602 (51.3%) were identified as methicillin-sensitive and 1,923 (37.9%) as MRSA; 545 (10.7%) had no susceptibility test results reported.

Although several statistically significant differences in facility characteristics were found in univariate analyses, the best candidate model for facility *S. aureus* bloodstream infection incidence indicated that *S. aureus* bloodstream infection risk was most strongly associated with patient vascular access type: compared with fistula access, CVC and graft or other had approximately six times (aRR = 6.2; 95% CI = 5.7–6.7) and approximately two times (aRR = 2.2; 95% CI = 2.0–2.4) higher risk, respectively (Table 1). Facility characteristics with statistically significant associations with *S. aureus* bloodstream infection incidence included any hospital affiliation (aRR = 1.5; 95% CI = 1.3–1.8), not being part of a chain of dialysis centers (aRR = 1.4; 95% CI = 1.2–1.7), not having a written antibiotic use policy (aRR = 1.3; 95% CI = 1.1–1.4), and location of the facility in an area with a higher proportion of persons aged ≥65 years (aRR = 1.4; 95% CI = 1.2–1.6; between highest quartile compared with lowest quartile) (Table 1).

**TABLE 1:** Independent factors associated with dialysis-associated *Staphylococcus aureus* bloodstream infection incidence* — National Healthcare Safety Network, United States, 2020

Characteristic	Likelihood ratio test^†^		aRR (95% CI)	p-value
	Chi-square	p-value		
**Vascular access type^§^**				
Central venous catheter	2,090.2	<0.001	6.2 (5.7–6.7)	<0.001
Graft or other			2.2 (2.0–2.4)	<0.001
Fistula			Ref	—
**Location/Hospital affiliation^¶^**				
Hospital**	113.0	<0.001	1.5 (1.3–1.8)	<0.001
Freestanding			Ref	—
**Member of group or chain of dialysis centers^¶^**				
No	111.7	<0.001	1.4 (1.2–1.7)	<0.001
Yes			Ref	—
**Written antibiotic use policy^¶^**				
No	35.1	<0.001	1.3 (1.1–1.4)	<0.001
Yes			Ref	—
**Quartile of % of persons aged ≥65 yrs^††^**				
Quartile 4: 75–100 (highest)	54.3	<0.001	1.4 (1.2–1.6)	<0.001
Quartile 3: 50–74			1.3 (1.2–1.5)	<0.001
Quartile 2: 25–49			1.3 (1.2–1.4)	<0.001
Quartile 1: 0–24 (lowest)			Ref	—

**Abbreviations:** aRR = adjusted rate ratio; ATSDR = Agency for Toxic Substances and Disease Registry; NHSN = National Healthcare Safety Network; Ref = referent group; SVI = Social Vulnerability Index.

* Negative binomial regression was used to fit this multivariate model.

**^†^** Likelihood ratio test evaluates whether a factor is statistically significantly associated with *Staphylococcus aureus* bloodstream infection incidence. Additionally, Akaike and Bayesian information criteria and Wald and likelihood ratio chi-square tests were used to evaluate model fit. The best candidate model was validated using bootstrap resampling methods and independently assessed by two separate analysts.

^§^ Source of data was NHSN event surveillance.

^¶^ Source of data was NHSN Outpatient Dialysis Center Practices Survey. https://www.cdc.gov/nhsn/forms/57.500_outpatientdialysissurv_blank.pdf

** Location could be a hospital or a freestanding location owned by a hospital.

^††^ Source of data was CDC/ATSDR SVI.

**FIGURE 1:**
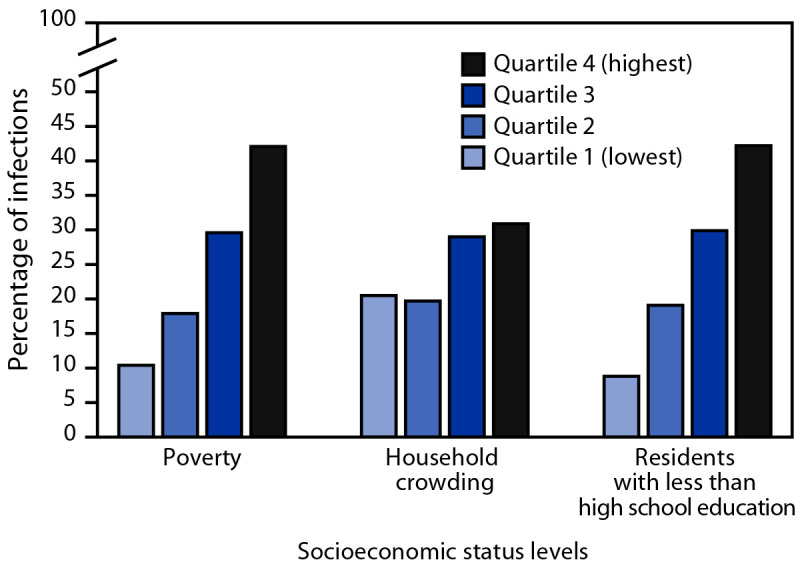
Percentage distribution of *Staphylococcus aureus* hemodialysis bloodstream infections among adult hemodialysis patients, by socioeconomic status levels of U.S. Census Bureau tracts of residence — Emerging Infections Program, United States, 2017–2020

**EIP.** During 2017–2020, 2,800 *S. aureus* bloodstream infections among hemodialysis patients were reported in the EIP surveillance areas. The overall annual *S. aureus* bloodstream infection rate was 100 times higher in hemodialysis patients (4,248 of 100,000 person-years) than among adults not on hemodialysis (42 of 100,000 person-years). U.S. Census Bureau tracts with lower SES factors accounted for disproportionately higher proportions of hemodialysis-associated *S. aureus* bloodstream infections (Figure 1). For example, 42.1% of *S. aureus* bloodstream infections among patients on hemodialysis occurred in tracts in the highest quartile of population proportion living below the poverty level, versus 10.4% in tracts in the lowest poverty quartile; similar distributions of *S. aureus* bloodstream infections according to crowding and educational levels also occurred. The overall population for the surveillance area was distributed relatively equally across tract-based quartiles for each of the SES factors examined.

**FIGURE 2:**
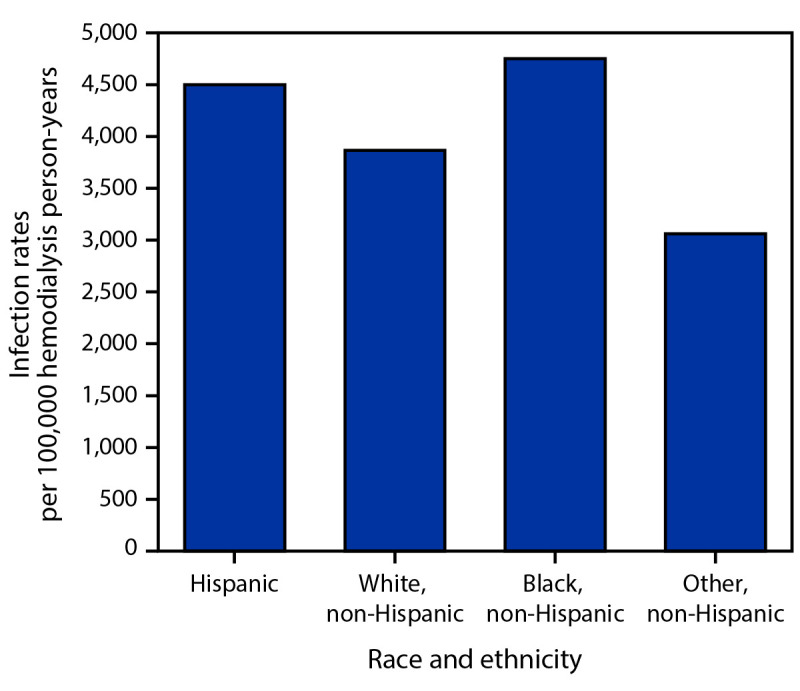
*Staphylococcus aureus* bloodstream infection rates* per 100,000 hemodialysis person-years, by race and ethnicity^†^ — Emerging Infections Program, United States, 2017–2020 * Unadjusted rates presented. ^†^ Race and ethnicity were categorized as non-Hispanic Black or African American (Black), Hispanic or Latino (Hispanic), non-Hispanic White, and non-Hispanic other (includes patients with more than one race recorded).

Unadjusted rates of *S. aureus* bloodstream infection were higher among Black and Hispanic hemodialysis patients (Figure 2) (Table 2). Other variables with higher rates included male sex, younger age groups, CVC access, and specific surveillance sites. The highest rates occurred among Black patients aged 18–49 years; 65% of bloodstream infections in this age, race, and ethnicity subgroup involved CVCs, which represented the highest prevalence of CVC use among the age, race, and ethnicity groups with bloodstream infections (range = 29%–65%). Multivariable analyses demonstrated that Hispanic ethnicity (aRR = 1.4; 95% CI = 1.2–1.7), male sex (aRR = 1.2; 95% CI = 1.1–1.4), younger age groups (patients aged 18–49 years [aRR = 1.7; 95 CI = 1.5–1.9] and patients aged 50–64 years [aRR = 1.2; 95% CI = 1.1–1.4] compared with patients aged ≥65 years), and specific sites were independent factors associated with higher bloodstream infection rates. However, CVC access had the strongest effect of all factors assessed. Black patients did not have significantly elevated risk when accounting for access type, age, sex, and EIP site. The proportion of hemodialysis patients who received a CVC was higher among White patients (23.1%) and Black patients (20.8%) than among Hispanic patients (13.9%).

**TABLE 2:** *Staphylococcus aureus* bloodstream infections associated with hemodialysis — Emerging Infections Program,* United States, 2017–2020

Characteristic	Univariate analysis			Multivariable analysis	
	No. of *S. aureus* bloodstream infections	No. of patient-years	Unadjusted rate^†^	aRR^§^ (95% CI)	p-value
**Age groups, yrs^¶^**					
18–49	736	11,848	6,212	1.7 (1.5–1.9)	<0.001
50–64	993	22,312	4,451	1.2 (1.1–1.4)	<0.001
≥65	1,071	31,758	3,372	Ref	—
**Sex**					
Female	1,112**	28,239	3,938	Ref	—
Male	1,685	37,679	4,472	1.2 (1.1–1.4)	<0.001
**EIP site**					
California	822	23,478	3,501	1.1 (0.9–1.4)	0.19
Connecticut	182	4,404	4,133	1.2 (1.0–1.5)	0.12
Georgia	393	6,218	6,320	2.0 (1.6–2.5)	<0.001
Maryland	741	15,022	4,933	1.4 (1.1–1.7)	0.003
New York	242	5,024	4,817	1.3 (1.0–1.6)	0.05
Tennessee	183	4,190	4,368	1.2 (1.0–1.5)	0.12
Minnesota	237	7,582	3,126	Ref	—
**Race and ethnicity^††^**					
Black, non-Hispanic	1,509	31,762	4,751	1.1 (0.9–1.2)	0.40
Hispanic	321	7,122	4,500	1.4 (1.2–1.7)	<0.001
White, non-Hispanic	687	17,764	3,866	Ref	—
Other, non-Hispanic	284	9,270	3,061	1.0 (0.8–1.2)	0.92
**Vascular access types^§§^**					
Central venous catheter	1,444	11,963	12,071	4.3 (3.9–4.8)	<0.001
Fistula or graft	1,303	48,631	2,679	Ref	—
**Total**	**2,800**	**65,918**	**4,248**	**—**	**—**

**Abbreviations:** aRR = adjusted rate ratio; EIP = Emerging Infections Program; Ref = referent group; *S. aureus* = *Staphylococcus aureus*.

* EIP data for January 1, 2017–December 31, 2020.

^†^ Per 100,000 hemodialysis person-years.

^§^ Adjusted for age, race and ethnicity, sex, vascular access type, and EIP site as appropriate.

^¶^ The median age of patients with hemodialysis *S. aureus* bacterial infection was 60 years (IQR = 49–70 years); the median age of those aged 18–49 years was 41 years.

** Sex was unknown for three patients.

^††^ Race and ethnicity were categorized as non-Hispanic Black or African American (Black), Hispanic or Latino (Hispanic), non-Hispanic White, and non-Hispanic other (includes patients with more than one race recorded). Race and ethnicity case counts are averaged over 10 imputations to account for missing values of race and ethnicity. The total sums to >2,800 because of rounding.

^§§^ Fifty-three cases had unknown vascular access type. The denominator includes 5,324 hemodialysis patient-years with unknown vascular access type.

## Discussion

Although hemodialysis bloodstream infections have decreased since 2014 with the widespread implementation of evidence-based prevention strategies in dialysis facilities (e.g., staff and patient education, CVC care practices, observations of infection prevention practices, and surveillance for infections), *S. aureus* bloodstream infections remain an important cause of morbidity in hemodialysis patients, with rates 100 times higher in hemodialysis patients than among adults not on hemodialysis during 2017–2020. Although vascular access type was the factor most strongly associated with *S. aureus* bloodstream infections, disparities by race, ethnicity, and SES were also observed.

Although it is well established that race, ethnicity, and social determinants of health affect the development of ESKD and treatment options (*1*–*3*), how they relate to dialysis-related infection risk has not been as well described. In this study, the higher unadjusted *S. aureus* bloodstream infection rates observed in Black and Hispanic patients support the higher infection risk described in other published reports (*13*,*14*). However, whereas higher crude rates were observed in Black patients in the current study, race was not a statistically significant factor in multivariable analyses, suggesting the higher unadjusted rate might be mediated by other factors; in contrast, Hispanic ethnicity was independently associated with a 40% higher risk for *S. aureus* bloodstream infection. Although the effect of insurance status and lower SES could not be analyzed in the multivariable model in this report, disproportionately higher numbers of hemodialysis patients with *S. aureus* bloodstream infections lived in U.S. Census Bureau tracts with higher poverty, more household crowding, and lower education levels. These findings suggest that Black and Hispanic dialysis patients have higher rates of *S. aureus* bloodstream infections, and that lower SES might also be related to development of *S. aureus* bloodstream infections.

Although CVC vascular access, a known major risk factor for hemodialysis bloodstream infections (*11*) was most strongly associated with *S. aureus* bloodstream infections independent of race, ethnicity, and SES, potentially important associations between race and ethnicity and vascular access type used should also be considered. For example, recent national data suggest that initiation of hemodialysis with a CVC does not vary substantially by race, ethnicity, or SES (*1*), although other studies have shown associations among Black race, Hispanic ethnicity, poverty, insurance status, and shorter duration of pre-ESKD care with lower initiation with fistula (*16*–*18*). In the current study, despite having a 40% higher risk for *S. aureus* bloodstream infections, EIP Hispanic hemodialysis patients had a lower proportion of CVC use, which along with recent national data (*1*), suggest that CVC use is unlikely to be the only factor mediating this increased *S. aureus* bloodstream infection risk. Duration of CVC use might also be important; one study found that Black and Hispanic incident hemodialysis patients aged ≥65 years have longer use of CVC access compared with White patients, with Black patients spending on average approximately 40 more days on CVC, and Hispanic patients and those of other races spending 20–30 more days (*19*). Other mediating factors might include how patients with CVCs are educated about CVC care or what resources are available for such care. Although more data are needed to better define these factors, further reducing rates of CVC use among those at most risk is an important step in reducing *S. aureus* bloodstream infections.

The complex relationships among age, race and ethnicity, social determinants of health, and hemodialysis-associated infection risk warrant additional study. In particular, strengthening hemodialysis bloodstream infection surveillance to more comprehensively assess social determinants of health would improve understanding of risk and address some of the limitations of this report, which is subject to at least two. First, analyses of 2020 NHSN facility–reported bloodstream infection data relied on linkage with ecologic 2018 SVI data that were not patient-specific and could not be summarized below the county level. This limited the strength of conclusions that could be drawn from small but statistically significant associations of bloodstream infection rates with facility characteristics such as location in areas with higher percentages of older adults. Similarly, it is unclear whether the associations observed between bloodstream infection rates and dialysis facility affiliation or having a written antibiotic use policy are related to facility organizational structure and staffing, reporting practices, or other infection control policies. Second, and in contrast to NHSN analyses, EIP data were available on patient age, race, and ethnicity, but bloodstream infection rates by SES factors could not be calculated because U.S. Census Bureau tract–level denominator data were unavailable. Instead, the number of bloodstream infections by tract quartiles for different SES factors were calculated. These and other differences between NHSN and EIP surveillance design, including differing case ascertainment methodologies, reflect different primary surveillance purposes that have led to relatively lower bloodstream infection case estimates in NHSN (*20*). Overall, improved surveillance through closer linkage of existing relevant data sources and more granular surveillance data capture (e.g., patient-level information about access type, race and ethnicity, and SES), especially through automated reporting, would provide additional insight to address health disparities through specific public health interventions without increasing reporting responsibilities for health care providers.

Because disparities can affect ESKD development, access to treatment options, and risk of hemodialysis bloodstream infections, a comprehensive approach to preventive care that recognizes racial, ethnic, and socioeconomic disparities is needed. This approach could include continued efforts to prevent and improve management of underlying conditions such as diabetes and hypertension, improved access to care for prevention and early recognition and treatment of chronic kidney disease, and increased availability of optimal treatments for ESKD, particularly in areas of lower SES. In addition, the use of cultural- and language-appropriate patient education might help patients in the care of dialysis access and infection prevention, which might be especially relevant for Hispanic patients given the higher *S. aureus* bloodstream infection risk observed in Hispanic persons (*3*). Given the importance of CVC use as a risk factor, further investigation of the determinants of CVC use and duration including possible disparities by population subgroups is needed and could further minimize CVC use and address possible barriers to lower-risk access types. Regardless, education and implementation of established best practices to prevent bloodstream infections^†††^ are critical to protecting the entire hemodialysis patient community, including those most at risk.

**Summary**

**What is already known about this topic?**

Racial and ethnic minorities are disproportionately affected by end-stage kidney disease (ESKD), and patients on dialysis are at increased risk for *Staphylococcus aureus* bloodstream infections. Hemodialysis access type is a well-established risk factor for bloodstream infections, with central venous catheters having the highest associated risk.

**What is added by this report?**

Although vascular access type was the major risk factor for hemodialysis-associated *S. aureus* bloodstream infections, race, ethnicity, and socioeconomic factors also affected infection rates and distribution, with Hispanic or Latino ethnicity as an independent risk factor.

**What are the implications for public health practice?**

Health care providers should prioritize prevention and optimized treatment of ESKD, identify and address barriers to lower-risk vascular access placement, and implement established best practices to prevent bloodstream infections.

## Footnotes

*
https://www.cdc.gov/healthequity/whatis/

†
https://www.cdc.gov/dialysis/pdfs/BSI-NHSN-2014to2019-508.pdf

§ 45 C.F.R. part 46.102(l)(2), 21 C.F.R. part 56; 42 U.S.C. Sect. 241(d); 5 U.S.C. Sect. 552a; 44 U.S.C. Sect. 3501 et seq.

¶
https://www.cdc.gov/nhsn/dialysis/event/index.html

** *S. aureus* bloodstream infection incidence was modeled using forward stage-wise negative binomial regression. Akaike and Bayesian information criteria and Wald and likelihood ratio chi-square tests were used to evaluate model fit. The best candidate model was validated using bootstrap resampling methods and independently assessed by two separate analysts. Univariate analyses were also performed using the model.

†† The SVI data set included 15 individual vulnerability subthemes, four summary themes, and one overall vulnerability index score. The method of SVI scoring involves ranking U.S. counties and assigning each one a percentile, ranging from 0 to 100, where a higher value represents greater vulnerability. SVI variables were assessed as quartiles, and all other continuous variables were assessed as deciles, quintiles, quartiles, tertiles, and at the median.

§§ Race and ethnicity were categorized as Black, Hispanic, White, and other (which includes patients with more than one race recorded). Missing race (255, 9.1%) and ethnicity (310, 11.0%) information were imputed using the respective proportions of Black, Hispanic, and White persons in the U.S. Census Bureau tract population where the patient lived and the distribution of race and ethnicity among dialysis patients with known race and ethnicity by age group, sex, and county; 10 imputed data sets were created. Descriptive results involving race and ethnicity report the mean aggregated across all 10 imputation data sets; for multivariable modeling, the SAS MIANALYZE procedure was used to incorporate results from modeling the 10 imputation data sets.

¶¶ Measures of SES included the following: 1) percentage of population living below the poverty level, 2) percentage of households with crowding (more than one occupant per room), 3) percentage of adults aged ≥25 years with less than a high school education, using 2015–2019 (for 2017–2019 cases) or 2016–2020 (for 2020 cases) 5-year U.S. Census Bureau American Community Survey data (https://www.census.gov/programs-surveys/acs). U.S. Census Bureau tracts under surveillance were grouped by quartile for each measure of SES factors, and the number of *S. aureus* bloodstream infections occurring in tracts within each quartile of SES factors was described.

*** USRDS data were requested from and provided by USRDS (https://usrds.org). U.S. Census Bureau data were obtained from National Center for Health Statistics bridged-race vintage postcensal files. https://www.cdc.gov/nchs/nvss/bridged_race.htm

†††
https://www.cdc.gov/dialysis/prevention-tools/core-interventions.html
